# The diversity and evolution of ecological and environmental citizen science

**DOI:** 10.1371/journal.pone.0172579

**Published:** 2017-04-03

**Authors:** Michael J. O. Pocock, John C. Tweddle, Joanna Savage, Lucy D. Robinson, Helen E. Roy

**Affiliations:** 1 Centre for Ecology & Hydrology, Maclean Building, Benson Lane, Crowmarsh Gifford, Wallingford, Oxfordshire, United Kingdom; 2 Angela Marmont Centre for UK Biodiversity, The Natural History Museum, London, United Kingdom; East China Normal University, CHINA

## Abstract

Citizen science—the involvement of volunteers in data collection, analysis and interpretation—simultaneously supports research and public engagement with science, and its profile is rapidly rising. Citizen science represents a diverse range of approaches, but until now this diversity has not been quantitatively explored. We conducted a systematic internet search and discovered 509 environmental and ecological citizen science projects. We scored each project for 32 attributes based on publicly obtainable information and used multiple factor analysis to summarise this variation to assess citizen science approaches. We found that projects varied according to their methodological approach from ‘mass participation’ (e.g. easy participation by anyone anywhere) to ‘systematic monitoring’ (e.g. trained volunteers repeatedly sampling at specific locations). They also varied in complexity from approaches that are ‘simple’ to those that are ‘elaborate’ (e.g. provide lots of support to gather rich, detailed datasets). There was a separate cluster of entirely computer-based projects but, in general, we found that the range of citizen science projects in ecology and the environment showed continuous variation and cannot be neatly categorised into distinct types of activity. While the diversity of projects begun in each time period (pre 1990, 1990–99, 2000–09 and 2010–13) has not increased, we found that projects tended to have become increasingly different from each other as time progressed (possibly due to changing opportunities, including technological innovation). Most projects were still active so consequently we found that the overall diversity of active projects (available for participation) increased as time progressed. Overall, understanding the landscape of citizen science in ecology and the environment (and its change over time) is valuable because it informs the comparative evaluation of the ‘success’ of different citizen science approaches. Comparative evaluation provides an evidence-base to inform the future development of citizen science activities.

## Introduction

Citizen science is the ‘intentional involvement, in a non-professional capacity [i.e. as volunteers], of people in the scientific process…’ [1] and it has been rising in profile over the past decade [2–4]. It is increasingly seen by policy-makers, as well as scientists, as a cost-effective method for monitoring and research [5–9]. This is especially so in ecological and environmental research [10,11] where citizen science has a long history [1,12,13]. Records from volunteers have supported data-intensive science and monitoring at scales (spatially, temporally or simply in terms of volume of data) that are impractical or too costly to achieve without participation of volunteers, and this has been supported by a range of approaches to ensure data quality is adequate for the intended purpose [14]. The outputs have also been used for conservation benefits [12,15] and environmental protection [16,17].

One of the defining aspects of citizen science is that the focus is not simply on data, but that it is simultaneously a way of engaging people with science [2,12,18], which is also a valuable activity in its own right [19]. Often volunteers are primarily involved with data collection [2], the classification of records [20] and/or problem solving [21,22], but, depending on the project, people can also participate in the generation of research questions and project design [23], disseminating results and acting upon the findings [24]. Involving people in the process (not just the outputs) of scientific research can support increased scientific literacy through informal education [25], the democratization of science [7] and the development of public policy [26].

The term ‘citizen science’ is one that unifies these many different activities in a single field of practice [27] but in doing so it could be possible to overlook the remarkable diversity of approaches and methods across projects. These approaches are generalizable summaries of *how* a project was undertaken, and so they are different to *why* a project was undertaken (its goals and focus) or its success. Describing the diversity of citizen science approaches is therefore important and is a precursor to any comparative evaluation of projects: whether comparing science and engagement outputs of similar activities, or compare-and-contrasting the success of different approaches. Better comparative evaluation is necessary to provide an evidence-base to inform the future development of citizen science activities.

Previously there have been several studies seeking to describe diversity in citizen science; these descriptions have been based on expert judgement [7,23,26,28–30] or quantitative analysis of a relatively small number of projects [24,31,32]. However a large-scale, quantitative analysis of citizen science approaches has thus far been lacking (though see [11] for an analysis of outputs from biodiversity citizen science).

Our aim in this project was to undertake a thorough and quantitative assessment of the diversity of approaches of citizen science projects in ecology and the environment. We also aimed to assess how the diversity has changed over time. Specifically we expected that, with the growing interest in citizen science, its diversity would have increased over time and so we assessed both its ‘emerging diversity’ (the diversity of projects begun in a specified time period) and its ‘accumulated diversity’ (the diversity of all projects active in each time period, thus including active projects which were begun in previous time periods).

## Methods

### Search for citizen science projects

To undertake a comprehensive search for citizen science projects in ecology and the environment (land, water and air) in a repeatable, standardized way we undertook an internet search using the Google search engine (www.google.com) and the terms “citizen science”, “take part AND (nature OR environment)”, “volunteer-based monitoring”, “public participation in scientific research” and “participatory science”. Searches were conducted in July 2012 and updated January 2014. Cookies were cleared on the internet browser before searching. We followed the first 100 links from each search and all the links that they contained and listed all citizen science projects. In doing this we included (but were not restricted to) major citizen science directories, i.e. Citizen Science Central (http://www.birds.cornell.edu/citscitoolkit/projects), Scistarter (https://scistarter.com/), CitSci (http://citsci.org), Scientific American (http://www.scientificamerican.com/citizen-science/) and UK Environmental Observation Framework (http://www.ukeof.org.uk/catalogue). The breadth of our search terms meant that we could identify projects and programs that fit the definition of citizen science but do not necessarily self-identify as citizen science. We noted all potential ecological and environmental citizen science projects (including projects about the atmosphere, but excluding astronomy and human health) to produce a candidate list of 720 projects.

We visited the websites, or searched for additional information on all projects in the candidate list to produce a final list of 509 projects (having excluded 162 projects that were primarily about education and communication or conservation volunteering, and 49 projects about which we could find no further information). Some projects were comprised of different activities; where they were promoted as entirely separate activities (e.g. the audiences, methods and project branding were different to each other, leading to separate routes of promotion), we considered them separately. There were several types of project that were similar to each other (for example, regional water quality monitoring projects in the USA or taxon-specific biological recording schemes in Britain) but these were treated as individual projects.

The process for discovering and selecting projects was as inclusive and repeatable as possible, but we accept that relying on internet searches would have led to under-sampling of, and potential bias in, the types of project that were included in our analysis. A specific challenge is that the term ‘citizen science’ has only been popularized since the late 2000s, even though volunteers have been involved in a wide range of projects for much longer than this [1,2,13]. We expect that the following types of projects may have been under-sampled by our approach: (1) small-scale projects fitting the definition of citizen science although not defining themselves as such, (2) projects in non-English speaking communities (although some of the projects of which we were aware that are operating in languages other than English were discovered through the internet searches), (3) projects that do not have websites, (4) projects that have finished (especially those that ran in the decades prior to websites), and (5) projects not using the term ‘citizen science’ or ‘volunteer’.

### Attributes of citizen science projects

We scored each project for 32 attributes which described the approach of the project from publicly-available information, i.e. how the project was undertaken. The attributes included details about protocols, supporting resources, data accessibility, modes of communication and project scale (Table 1; Table A in S1 File) [33]. We included start and finish date of the project as supplementary attributes. These attributes were selected to describe the different citizen science approaches as fully as possible. We did not categorise why a project was undertaken, because we considered that any description of project goals is subjective and hard to categorise unambiguously, or project success, because success is multifactorial and needs to be evaluated against project-specific criteria. We also described four more supplementary variables: the main subject, physical domain, purpose and organisers of projects (Appendix A in S1 File). We did not use these supplementary variables in the main analysis because they described the specifics of the project, rather than providing a generalizable explanation of how the project was undertaken. An initial list of attributes was created by MJOP and HER and were tested and refined by the authorship on a subset of example projects to ensure they were as comprehensive and as robustly defined as possible. Each project was then scored once (by MJOP or JS) and all were subsequently reviewed by MJOP with minor changes made to scores, where necessary, to ensure consistency (see [11] for a similar approach). Websites with information on the projects were visited shortly after the initial internet searches were undertaken and all projects that were scored in 2012 were reviewed in 2014.

**Table 1:** Summary description of the first three multivariate axes and the 24 (out of 32) attributes correlated to these factors.

	Axis 1	Axis 2	Axis 3
Summary description of the multivariate axis (negative to positive extremes)	Systematic monitoring (-ve) → Mass participation (+ve)	Simple approaches (-ve) → Elaborate approaches (+ve)	Entirely computer-based (-ve) → Hands-on participation (+ve)
Support via personal training	**-0.69**		
Sites are self-selected	**+0.66**		
Snapshots are sufficient	**+0.65**		
Repeat visits required	**-0.62**		
Special equipment required	**-0.62**		
Geographic scope of project	**+0.57**		
Different types of data questions	-0.46	**+0.51**	
Routes to engagement: personal invitation	-0.46		
Best quality of data	-0.42	**+0.58**	
Support in advance	-0.40		
Routes to engagement: smartphone	+0.36		+0.32
Entirely computer-based	+0.36		**-0.81**
Type of record: photo	+0.35		+0.38
Availability of data: in real time	+0.34	+0.34	+0.38
Routes to engagement: email	-0.32		
Type of record: physical sample	-0.31		
Support via supporting material		**+0.52**	
Routes to engagement: website		+0.47	
Background context		+0.48	
Targetted at school children		+0.44	
Availability of data: to view		+0.42	+0.33
Registration required		+0.36	-0.37
Support via online media		+0.34	
Type of record: score		+0.31	
Type of record: location			**+0.77**

Only correlations with *r*>0.3 are shown and those with *r*>0.5 are in bold; full results in Table C in S1 File. For the correlations, positive/negative values indicate that the attribute is more/less likely (for binary attributes) or increases/decreases (for ordinal attributes) towards the positive end of the axis.

Scoring project attributes in this way has the advantage that all of the projects can be assessed consistently. However, it does rely on the accuracy of publicly-available information and we accept that our scoring of a project from the information gathered from publicly-available websites may not agree with the perceptions of the project organisers, although it should reflect the perspective of potential recruits to the project. We also acknowledge that projects and their websites may have changed since we undertook the scoring. Overall, therefore, we do not propose that our scoring is viewed as an indisputable summary of an individual project, but we are confident that the overall results were not strongly influenced by these minor discrepancies. Our methods allowed us to assess all projects in the database. Relying on surveys of project organisers results in a smaller sample that may not be representative (due to systematic variation in people’s enthusiasm to respond to surveys, e.g. based on their perception of their project’s success) which could result in a biased sample. However surveys of project organisers do allow attributes such as organisers’ motivation, the project’s ‘success’ and ongoing use of data to be considered [11,24,31,32].

### Statistical analysis

All analyses were carried out with R 3.2.5 using packages as listed below.

We assessed the cumulative rate of increase in the number of projects over time. Because this rate was unlikely to be constant over time, we used a segmented regression [34] to identify timepoints when the rate changed. We used data from the year 1850 (3 projects) to 2013 with the package ‘segmented 0.5–0.0’ [35] and modelled the trend with 1, 2 and 3 breakpoints. Only 2% of the projects could not be given a start date; these were excluded from this analysis, but they were allocated to one of the four general date classes as used later.

In order to describe the landscape of the diversity of citizen science in ecology and the environment, we used multiple factor analysis (MFA) to reduce the 32 project attributes to a smaller number of factors using the package ‘factoMineR’ [36]. MFA is a multivariate analysis technique based on principal components analysis (PCA), but permits both nominal and hierarchical data to be included as both explanatory and supplementary variables [36,37]. The result of MFA is a series of factors (orthogonal axes) that each explain a decreasing proportion of the total variance in the data.

It is not straightforward to decide the optimum number of factors which are sufficient to explain the variation in the data and various ‘rules of thumb’ have been proposed [38], e.g. all factors with eigenvalues greater than 1 (6 factors for our results), an estimate of where the ‘scree plot’ of eigenvalues against factors changes in slope (6 or 7 factors), the point at which the scree plot stops substantially decreasing (4 factors), or the number of factors that are highly correlated with multiple attributes, i.e. they are true multivariate axes (2 or 3 factors; Table C in S1 File). Taking a conservative approach, we concluded that three factors were sufficient to usefully summarise the variation in citizen science projects, however each additional axis explained further variation (Fig. A in S1 File).

With the MFA results, we tested two hypotheses. Firstly, we tested whether projects could be grouped into distinct types by statistically testing for clusters in the multivariate space, or whether variation was continuous. We clustered the projects based on the first three factors of the multifactor analysis with hierarchical clustering (‘hclust’ in the ‘stats’ library in R 3.2.5) using the unweighted pair-group method using arithmetic averages (UPGMA) based on the Euclidian differences between projects (for the three MFA factors) [37]. This differentiated between the two paradigms for describing diversity: typological, i.e. the classification into discrete types as in Linnaean biological taxonomy and applied to linguistics and organizational science [39,40], and continuous, i.e. position along continuums in multivariate space, e.g. as has been used to describe variation in human personality ‘types’ [41].

Secondly, we tested whether citizen science in ecology and the environment and its diversity has changed over time. We considered four time periods: before 1990s, during 1990s, during 2000s and from 2010–2013 and assessed the changing diversity of projects by considering projects that began in each period (the ‘emerging’ diversity) and projects active during each period (the ‘accumulated’ diversity).

The diversity of citizen science projects in ecology and the environment in each time period was shown by plotting their positions according to the first two axes of the MFA and it was quantified using two approaches. The first approach was using kernel methods to estimate the ‘utilization distribution’ [42] of the points. The utilization distribution defines a region (or regions, because the distribution can be discontinuous) enclosing a certain proportion of the points (according to a user-specified threshold value), so a larger utilization distribution indicates greater citizen science diversity. Utilization distribution was calculated with ‘adehabitatHR 0.4.11’ [43]. Using a higher threshold value gives an estimate of the core distribution, and using a lower probability value gives an estimate of the complete distribution.

The second approach was calculating the angular spread of points around the origin of the MFA. Points that were spread around the two-dimensional plot would result in a high angular spread, whereas points clustered in one segment would have a low angular spread. Angular spread was calculated as the angular deviation *s* = √(2(1-*r*)), where *r* is the length of the mean vector of all points included in the plot (i.e. the ‘resultant length’) [44], and was calculated with ‘circular 0.4–7’ [45]. This measure accounts for the angular position of the points, but takes no account of their distance from the origin in the MFA.

As well as assessing the diversity of projects within each time period, using these two complementary approaches, we also compared the distribution of points from each time period to the distribution of points from every other time period. As before, we did this in two ways. Firstly, we calculated the overlap between the utilization distributions of the two sets of points. A larger overlap indicates that the two distributions of points are more similar to each other than if the overlap was less. We quantified the volume of intersection between the full utilization distributions (the ‘VI index’) for the two distributions of points [46] with the package ‘adehabitatHR 0.4.11’ [43]. Secondly, we assessed the difference between the angular spreads of the two distributions. An increasing statistical significance between the two angular spreads indicates that the two spreads are increasingly different from each other. Statistical significance was based on Watson’s two sample test of homogeneity: U^2^, with critical values of U^2^ determined from tables in [47]).

## Results

### Overview of results

Our systematic search revealed 509 projects fitting the definition of environmental and ecological citizen science (S1 Dataset). The majority of projects were focused on biodiversity (77%) rather than the abiotic environment (e.g. water quality or atmospheric pollution), and for most (93%) volunteer involvement was limited to contributing data, rather than collaborative or co-created project development [23,29] (Fig. E in S1 File).

### The rate of increase in citizen science projects

**Fig 1:**
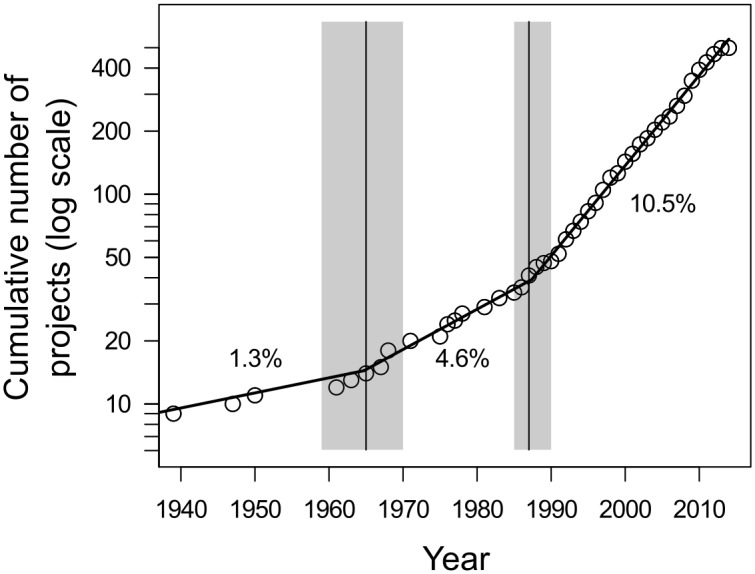
The rate of increase in the cumulative number of ecological and environmental citizen science projects as revealed by a systematic search. Black vertical lines indicate the estimated years when the rate of increase changed, as indicated by segmented regression, with 95% confidence intervals around these estimates indicated by grey rectangles (see Table B in S1 File).

We found that the cumulative number of projects discovered via our systematic search (most of which are still active) has increased exponentially by 10% per year consistently for over two decades (Fig 1). The segmented regression of the cumulative number of projects showed strong support for the presence of two break points, with increases in about 1962 and 1987 (Table B in S1 File), so while the number of projects we found is increasing exponentially over time, there was no evidence, so far, that this rate of exponential growth has increased since the late 1980’s.

### The diversity of citizen science projects

**Fig 2:**
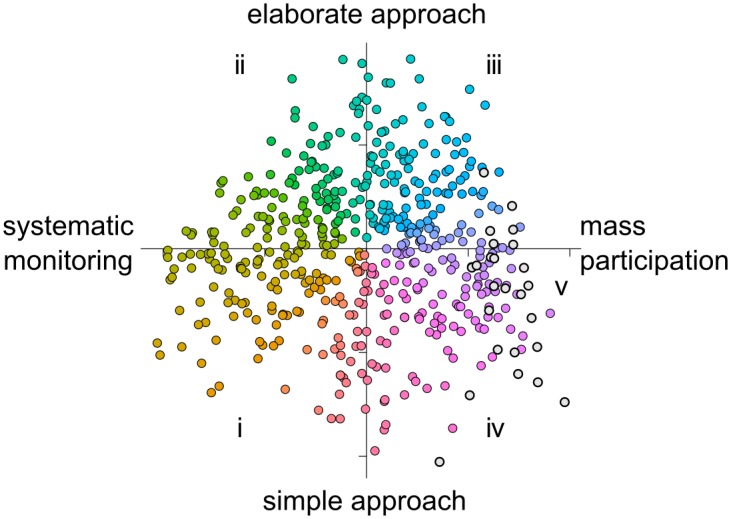
The landscape of citizen science described by a Multi-Factor Analysis (MFA) of 32 attributes of 509 citizen science projects in environmental and ecological science. The majority of projects (coloured points) are best described according to their position on the first two multivariate axes (the methodological approach: x axis, and the complexity of the approach: y axis). There is a separate cluster of computer-based projects (light grey points, labelled ‘v’) explained by their position on the third axis. The quarters of the plot and the grey points are labelled i-v for reference to Fig 3A & 3B. Points are coloured with the hue-chroma- luminesence colour scheme to avoid perceptual artefacts from the rainbow colour scheme [61].

Quantitative analysis of the project attributes with multi-factor analysis (MFA) revealed that projects are continuously distributed along the first two factors, which together explained 22% of the total variance (Fig 2, Figs A and B in S1 File). The two axes represent combinations of the individual project attributes (Table 1; Table C in S1 File). We summarised Axis 1 as the ‘methodological approach’ and varied from what we describe as ‘mass participation’ projects (that tended to be those in which anyone can get involved anywhere) to ‘systematic monitoring’ (that tended to require participation at pre-defined sites that are visited repeatedly and to require particular equipment e.g. binoculars or tape measures). We summarised Axis 2 as the complexity of the citizen science activity and varied from what we describe as ‘elaborate’ approaches’ (that tended to have complex protocols coupled with comprehensive supporting material and yield comparatively rich datasets) to ‘simple’ approaches (that tended to have little or no structured protocol, although they may require expertise such as species identification, and produced datasets with simple structures). We note that these descriptions do not signify degree of ‘success’: ‘success’ itself is multifactorial and should be judged according to the intended purpose of the project, the production of data and knowledge, and engaging people with science and research [48].

**Fig 3:**
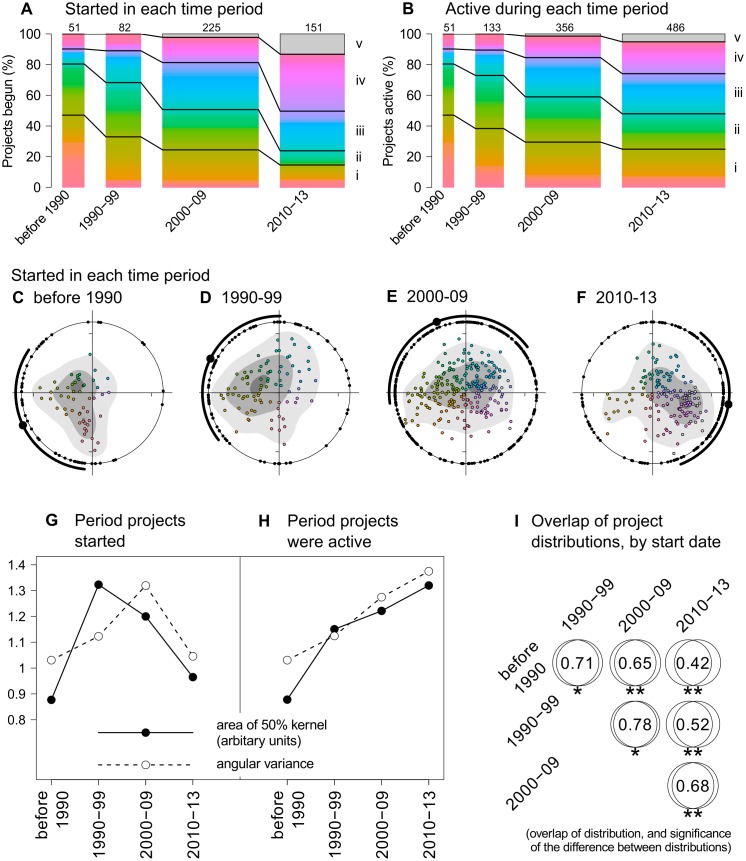
Changes in the landscape of citizen science over time, showing the emerging diversity (projects started in each period) and accumulated diversity (projects active in each period). A shifting focus of project instigation (A, C-F, I), despite a lack of consistent increase in citizen science science diversity (G) has resulted in the accumulated diversity of citizen science over time (B, H). Coloured bars (A,B) represent angular position (see Fig 2 for details). Bar width is relative to number of projects, and black lines indicate position of cartesian axes (with the four quarters and the separate cluster labelled i-v). The shifting focus over time (C-F) is revealed by kernels (dark, mid and light grey indicating 5, 50 and 90% kernels, respectively) and angular position (individual projects projected as black points on the circle; the large point and arcs showing angular mean ± deviation), which are summarised for emerging (G) and accumulated (H) diversity. Projects are becoming increasingly different over time as indicated by proportion of kernel overlap and Watson U^2^ test for differences in angular distributions (I: *<0.05; **<0.01).

In addition to these first two axes, the third axis (explaining 8% of the variation) separated a group of projects, which were entirely computer-based (Table 1, Fig. B in S1 File), from the remainder of projects. Computer-based projects have been described as ‘volunteered thinking’ [29], because they represent the crowd-sourcing of tasks requiring human interpretation, e.g. classifying images or sounds or solving problems in a game-style environment [15,29]. They are increasing in frequency (Fig 3A), presumably through the opportunities provided by increasing internet connectivity coupled with technological innovation.

Cluster analysis of the results of the first three axes clearly indicated that there were two distinct clusters and this differentiation was based mainly on position on the third factor (Fig. B in S1 File). (This result was identical when considering clustering with the results of the first four factors.) No further clustering was evident, so leading to the conclusion that the majority of projects were continuously distributed along the first two axes (the methodological approach and project complexity), and discontinuously distributed along the third factor (entirely computer-based).

### Change in the diversity of citizen science over time: The evolution of citizen science

There were three important changes in the landscape of citizen science in ecology and the environment over time (Fig 3). Firstly, the ‘emerging diversity’ of citizen science has not increased over time (Fig 3C–3F): this is the diversity of projects that *started* in each specific time period. This is shown by the fact that there is no consistent trend in the diversity of projects in each time period, whether assessed by the area of the utilization distribution or the angular spread of projects around the origin (Fig 3G). This was contrary to our initial expectation that citizen science in ecology and the environment is becoming more diverse.

Secondly, projects begun in each time period are different from each other and becoming more different as the time gap becomes greater (Fig 3I). In other words the ‘emerging diversity’ of citizen science has shifted over time, and this change has been directional. The evidence for this is the increased difference between projects as the time gap between the periods in which they started becomes greater (Fig 3I). What is occurring is that projects begun since 2000 tend towards mass participation rather than systematic monitoring and those begun since 2010 tend to be simple rather than elaborate approaches, plus there are more entirely computer-based projects (Fig 3A and 3C–3F).

Thirdly, and as a result of the first two changes, the diversity of projects which are active has increased over time (Fig 3H) because the type of projects started are changing over time but few projects that we discovered had stopped (92% were still extant at the time information about projects was collected). In other words, the diversity of citizen science in ecology and the environment appears to be accumulating over time (Fig 3B cp. 3A).

### Other attributes relating to the diversity of citizen science

We have described the key attributes relating to each of the three main factors in the analysis, thus allowing us to summarise these axes as the methodological approach (from systematic monitoring to mass participation) and its complexity (from simple to elaborate approaches), plus the third axis distinguishing entirely computer-based projects from the rest. However the correlated attributes add further detail to the description of these factors (Table 1).

Considering the first factor, projects that tended towards being ‘mass participation’ also tended to ask fewer questions of participants and did not require high data quality or scoring of observations (e.g. making counts or taking measurements), but did allow participation via smartphones. In contrast, projects that tended towards being ‘systematic monitoring’ were more likely to engage people via personal contact or email, to provide support in advance and to request physical samples. Mass participation projects were more likely to have a larger geographic scope, whereas systematic monitoring projects were more likely to have a smaller geographic scope.

Considering the second MFA axis, more elaborate projects tended to: (1) ask more types of question and request a higher quality of data (e.g. measurements and counts, compared to only recording presence); (2) provide supporting materials with detailed background information, including via online media (such as videos) and; (3) be website-based (because that technology is ideal for delivering complex information and capturing rich data). Projects targeted to school children tend to have more elaborate approaches.

The availability of the data was associated with all three axes of the MFA. Specifically, presenting data dynamically (e.g. in real time rather than in summary reports) was more likely for projects that were mass participation (rather than systematic monitoring) and elaborate (rather than simple) and less likely for entirely computer-based projects (compared to the remainder). Also simple projects and entirely computer-based projects were less likely to make data available to view and download at better resolution (e.g. full dataset, rather than data summaries or reports) than other approaches.

The full results of the MFA are available in an interactive webapp (https://shiny-apps.ceh.ac.uk/citizen_science_landscape/), created with Shiny in R, allowing users to enter attributes for their own project and explore the way variation in attributes changes the position of the activity in the landscape of citizen science.

## Discussion

### Citizen science is a diverse range of approaches

Citizen science projects have characteristics that unify them as a distinct method of inquiry [3,27], i.e. the collection of data and participation wider than just ‘professional scientists’. However, here we undertook the first large-scale and quantitative assessment of diversity in citizen science in ecology and the environment (cp. [11,24,32]) to describe the sheer diversity of approaches across 509 projects—from systematic monitoring to mass participation, from simple to elaborate approaches, and from being entirely computer-based to being physically hands-on. The number of projects we discovered via a systematic search compares favourably with a comment in 2012 that deemed 280 projects to “be close to the size of the population” [24] and a recent review of 388 biodiversity citizen science projects discovered through searches of directories of citizen science projects [11]. Strikingly, projects do not aggregate into clearly-defined clusters of citizen science approaches (apart from entirely computer-based projects, which are distinctly different from the remainder) but instead there is continuous variation according to two axes of variation: the methodological approach and its complexity. Therefore, citizen science in ecology and the environment is not a single approach, but nor is it a collection of distinct, clearly-defined approaches. It seems that any discrete ‘classification’ or ‘typology’ of citizen science is one that is imposed upon the diversity of citizen science, rather than being a natural explanation emerging from it. This explains why it is so challenging to create a detailed typology or classification of citizen science or succinctly provide guidance on selecting citizen science approaches [30]. In the past, simplified descriptions of citizen science approaches have been used and they can be helpful [7,23,26,28–30] but it must be remembered that any such typologies are simplifications of the incredible diversity of approaches.

Explaining the diversity of citizen science, as we have done for projects in ecology and the environment helps to elucidate the breadth of citizen science approaches (and show how they are related to other project attributes such as project purpose or the degree of participation; Appendix A in S1 File). Understanding this diversity should help future project organisers to consider the full range of opportunities when deciding the most appropriate approach for their needs [30], rather than being constrained by their own experience or preconceptions. Our data-led description of citizen science diversity is therefore complementary to existing expert-led classifications of citizen science approaches [23,28,29].

### Citizen science in ecology and the environment is changing over time

A striking result from this study is that there was no evidence that citizen science in ecology and the environment was becoming more diverse over time (at least, considering the projects *begun* in each time period). However, citizen science has changed over time, resulting in an accumulation in the diversity of citizen science approaches for projects that were *active* during each time period. We suggest three reasons why citizen science may be changing over time.

Firstly, changes in citizen science could be because there are bursts of innovation permitted by successive technological innovations, e.g. online databases, digital photography and smartphones (with integrated cameras, geo-location and internet connectivity). This is akin to adaptive radiations in biology that occur when there is ‘ecological opportunity’ (e.g. competitive release or key evolutionary innovations) [49]. We found that projects based on smartphones and entirely computer-based projects have been increasing since 2010, presumably since these technologies have become widely available.

However, technology can also constrain activities, e.g. smartphones are most effective when considering ‘simple’ rather than ‘elaborate’ approaches (because, despite their many advantages, they have small screens and accurate typing is not easy). Thus any projects developed with a particular technological innovation (e.g. a future example might be augmented reality games [50]) would be characterised by the same opportunities and constraints as each other. The risk is that new technologies could be used simply because they are novel, rather than because they are appropriate, and so the technology constricts innovation in citizen science.

Secondly, changes in citizen science could be because of changing societal and cultural acceptability (hence, fundability) of different types of projects. We found that projects started since 2000 tended to be mass participation, which fits with the recent focus on widening participation [6,7], as well as the benefits provided by the growth of the internet and communications technology. It could also be because of the desire to pursue novel approaches rather than replicate existing approaches. Based on our results on the evolution of citizen science to date, we anticipate that both technological innovation and societal acceptability will be important in influencing and shaping the future of citizen science.

Thirdly, advances in statistical approaches have meant that data collected in a less-structured way than can be more usefully analysed than was previously the case [51], especially when large amounts of data are available [16]. Therefore data from mass participation approaches have become more scientifically credible.

### Potential biases in our analysis

There are two potential sources of bias in our analysis: the process of discovering and selecting projects for analysis, and the process for obtaining information about each of the selected projects (see Methods for description of these potential biases).

Overall, we expect that any biases resulting from under-sampling different types of projects would have been strongest for projects that do not have websites (e.g. are extremely local or small-scale) and those that began before the term ‘citizen science’ was popularised. It is difficult to envisage a repeatable, standardised approach that would effectively capture information about these projects and, anyway, the growth of the use of the term ‘citizen science’ is intrinsically linked to the growth in citizen science activities and vice versa. We would therefore be cautious in assuming the reported number of citizen science projects is precise, but our reported trends in citizen science projects and variation across projects broadly reflects our experience.

We are also aware that ‘citizen science’ does not have a precise definition and the term has been retrospectively fitted to activities which have not defined themselves as ‘citizen science’ e.g. because they existed before the popularisation of the term. There are other types of activity that share attributes of citizen science e.g. ‘participatory monitoring’ [17,52,53], ‘farmer participatory research’ [54] and ‘biological recording’ [1]. The first two appear to be frequent in non-English speaking tropical communities, whereas British examples of biological recording are listed in the UK Environmental Observation Framework catalogue (http://www.ukeof.org.uk/catalogue), which was discovered during our systematic search. Our search was likely to be biased towards English-speaking and European countries and it will be interesting to see how citizen science activities develop, and have developed, elsewhere in the world.

The process for obtaining information about the projects was based on publicly-available information: it reflected the perspective of potential recruits to the project. We accept that it may not have reflected the expectations of project organisers or the experience of active participants and it restricted the attributes we could consider to classify projects (because important attributes such as amount of funding were rarely provided). However, our approach allowed us to score all projects in our dataset and to do so consistently.

Ultimately, we accept that our final set of projects and their attributes will have some biases, as is inevitable for any study such as this. However our search was standardised, repeatable and, we believe, generally representative of the current state of environmental and ecological citizen science. In particular, we do not believe that the inclusion of otherwise undetected projects would have substantially altered our results or conclusions, although further research in this area would be valuable.

### Future considerations and how understanding the ‘landscape of citizen science’ can support citizen science evaluation

The number of citizen science projects in ecology and the environment has, according to information obtained with our systematic search for projects, been increasing at an exponential rate. However, it was surprising that this rate has not increased since the late 1980s, despite the rising profile of citizen science during this time. It remains to be seen how citizen science will develop in the future. One hypothesis we have is that citizen science activities will begin to coalesce into distinct types of approaches, especially if there is greater sharing of best practice, as is currently recommended [3]. This could be expected due to bottom-up processes (if successful projects act as exemplars for subsequent projects) or top-down processes (if organizational or funding constraints cause methods to converge), as analogous to convergence in ecological communities [55]. Such a trend could be either positive (if it enhanced the success of future citizen science) or negative (if increased professionalization of citizen science caused risk-adverse decisions on funding and project design). Of course, the alternative hypothesis is that continued innovation in citizen science, and the utilization of diverse technological innovations, will continue to cause the diversification of citizen science. If funders lack an appreciation of the diversity of citizen science approaches then this could lead to a narrow and stereotyped view of citizen science (both by proponents and critics) that could limit the development of citizen science in ecology and the environment and we hope the results of this study reduce this risk.

Currently there is a strong agenda for ‘open science’, which links to both citizen science and ‘open data’ (i.e. data that are available to all and for all to use) [56]. It would seem that there is a strong moral and societal case for citizen science data to be openly accessible (because it was collected by volunteers) [57], although this is not always the case for volunteer-collected biodiversity data [58]. However, it is concerning that our results suggest that projects with simple approaches and entirely computer-based projects, both of which have increased in frequency since 2010 (regions iv and v in Figs 2 and 3A), actually tend to be *less* likely to make data available to view and to download than other types of project (Table 1). There are many reasons why this might be the case, e.g. participation via smartphones tends to facilitate data collection rather than data exploration (due to the limitations of screen size) or for entirely computer-based projects it is the interpretation of data, not data collection, which is crowd-sourced and the data themselves may not be the main motivation for participation in such projects [59]. Nonetheless it is important that project organisers develop clear strategies about openness of data that acknowledges current policy agendas, moral imperatives and participants’ motivations.

Practitioners increasingly need to evaluate the diverse outputs and impacts of citizen science (i.e. its ‘success’) in terms of scientific outputs [10], breadth and quality of engagement [25,60], impact on policy [26], and even monetary value of volunteer contributions [9,11,15]. Our findings about the current diversity of citizen science in ecology and the environment and its change over time, will support the comparative evaluation of citizen science because it enables the similarity of approaches to be quantified. This then allows projects and their outcomes to be assessed, whether comparing projects with similar approaches or contrasting those with different approaches. The crucial point is that our findings provide, for the first time, an objective way to define how similar or different projects are, based on the results of the multivariate analysis (Fig 2), and this method is available for use via a web app (https://shiny-apps.ceh.ac.uk/citizen_science_landscape/).

Overall, we believe that describing the diversity of citizen science in ecology and the environment will help practitioners be more informed about the range of approaches that are available when developing new citizen science activities, and will enable more rigorous comparative evaluation of citizen science projects, for the future benefit of this field of practice.

## Supporting information

S1 File Supplementary information containing: Table A. The 32 attributes (and associated subcategories) used for scoring the 509 projects. Table B. Results of the segmented regression of the increase in citizen science projects over time. Table C. The correlation of the individual attributes with the first seven factors of the multifactor analysis (MFA). Figure A. Assessment of the multifactor analysis (MFA) results according to (i) a scree plot of eigenvalues against factors and (ii) number of attributes correlated with factors. Figure B. The distribution of projects in the first three factors from the results of the multifactor analysis. Figure C. Variation in the kernel approach and calculation of angular position and deviation between the different time periods when considering projects according to (i) the time period in which they started (the ‘emerging diversity’) and (ii) the time periods in which they were active (the ‘accumulated diversity’). Figure D. The area of the distribution of projects according to the first two factors of the multifactor analysis for different probabilities of the kernel for the emerging and the accumulated diversity (solid and dotted lines, respectively). Appendix A. Summary of analysis of additional supplementary variables: main subject, physical domain, project purpose, degree of participation, type of project lead partner and number of project partners. This includes Table D and Fig. E.
(DOCX)

S1 Dataset The full results of the scoring of the 509 citizen science projects in ecology and the environment according to 32 attributes, time period of start and finish and six supplementary attributes as obtained from publicly-available information. (CSV)
