# Supplementary material for: The diversity and evolution of ecological and environmental citizen science
Source: PLoS One. 2017 Apr 3;12(4):e0172579. doi: 10.1371/journal.pone.0172579 (PMC5378328; doi:10.1371/journal.pone.0172579)

S1 File: Supplementary information for: The diversity and evolution of ecological and environmental citizen science: a quantitative assessment

**Authors:** Michael J.O. Pocock^1^*, John C. Tweddle^2^, Joanna Savage^1^, Lucy D. Robinson^2^, Helen E. Roy^1^

**Affiliations:** ^1^ Centre for Ecology & Hydrology, Maclean Building, Benson Lane, Crowmarsh Gifford, Wallingford, Oxfordshire OX10 8BB, UK. ^2^ Angela Marmont Centre for UK Biodiversity, The Natural History Museum, Cromwell Road, London, SW7 5BD, UK.

* Corresponding author. Email: [michael.pocock@ceh.ac.uk](mailto:michael.pocock@ceh.ac.uk) (MJOP)

**Table A.** The 32 attributes (and associated subcategories) used for scoring the 509 projects.

**Table B.** Results of the segmented regression of the increase in citizen science projects over time.

**Table C.** The correlation of the individual attributes with the first seven factors of the multifactor analysis (MFA).

**Figure A.** Assessment of the multifactor analysis (MFA) results according to (i) a scree plot of eigenvalues against factors and (ii) number of attributes correlated with factors.

**Figure B.** The distribution of projects in the first three factors from the results of the multifactor analysis.

**Figure C.** Variation in the kernel approach and calculation of angular position and deviation between the different time periods when considering projects according to (i) the time period in which they started (the ‘emerging diversity’: a repeat of Fig. 3C-F in the main text) and (ii) the time periods in which they were extant (the ‘accumulated diversity’).

**Figure D.** The area of the distribution of projects according to the first two factors of the multifactor analysis for different probabilities of the kernel to assess the emerging and the accumulated diversity.

**Appendix A.** Summary of analysis of additional supplementary variables: main subject, physical domain, project purpose, degree of participation, type of project lead partner and number of project partners. This includes Table D and Fig. E.

**Dataset S1.** The full results of the scoring of the 509 citizen science projects in ecology and the environment according to 32 project attributes, time period of start and finish and six supplementary attributes as obtained from publically-available information.

**Table A.** The 32 attributes (and associated subcategories) used for scoring the 509 projects. See Appendix A for analysis of additional supplementary variables.

| Attribute | Subcategory | Data type* | Options [number of projects] | Notes |
| --- | --- | --- | --- | --- |
| Start period |  | C | Before 1990; 1990s; 2000s; 2010s | A supplementary variable, not used in the multivariate analysis |
| Start year |  | O | Year | A supplementary variable, not used in the multivariate analysis |
| Finish period |  | C | Before 1990; 1990s; 2000s; 2010s | A supplementary variable, not used in the multivariate analysis |
| Geographic scope |  | O | Local; regional; national; international |  |
| Aims |  | O | None apparent [19]; vague [146]; clear [344] | This only refers to the aims that were made publicly available |
| Background context |  | C | Minimal [68]; some [199]; context-rich [242] | This only refers to the context that was made publicly available (training courses may provide additional context) |
| Protocol | Site selection by participant | B | Yes [368]; no [141] | Can the participant choose where to participate? |
|  | One-off snapshots sufficient | B | Yes [203]; no [306] | A ‘snapshot’ is a record that takes little time, e.g. less than a minute or two |
|  | Repeat visits required | B | Yes [196]; no [313] |  |
| Routes to engagement | Website | B | Yes [361]; no [148] | The routes to engagement are the routes in which participants can directly engage with the project, e.g. discovering more information and submitting data |
|  | Smartphone | B | Yes [62]; no [447] |  |
|  | Social media | B | Yes [26]; no [483] |  |
|  | Mail | B | Yes [98]; no [411] |  |
|  | Email | B | Yes [65]; no [444] |  |
|  | SMS | B | Yes [5]; no [504] |  |
|  | Personal invitation | B | Yes [92]; no [417] |  |
| Type of support | In advance | B | Yes [68]; no [441] |  |
|  | Personal training | B | Yes [157]; no [352] |  |
|  | Supporting material | B | Yes [350]; no [159] |  |
|  | Online media | B | Yes [23]; no [486] | Other media in addition to written documentation, e.g. videos |
| Target audience | School children | B | Yes [64]; no [445] |  |
|  | Experts | B | Yes [126]; no [383] | Experts are people who already have skills needed to participate (e.g. bird identification) |
| Registration required |  | B | Yes [267]; no [220] | Registration is required to participate and submit data |
| Entirely computer based |  | B | Yes [25]; no [484] |  |
| Different types of data questions |  | O | 1 [85]; 2-5 [248]; >5 [176] | This is about the different types of question, not the actual number of questions. It gives an indication of the complexity of the protocol. |
| Special equipment required |  | B | Yes [287]; no [222] | ‘Special equipment’ is anything that would need to be especially taken out, e.g. tape measure, binoculars |
| Type of record | Location | B | Yes [472]; no [37] | e.g. For species occurrence records |
|  | Score | B | Yes [466]; no [43] | Something in addition to a location, e.g. a count or measurement |
|  | Photo | B | Yes [142]; no [367] |  |
|  | Other media | B | Yes [17]; no [492] | e.g. Sound files, video |
|  | Physical sample | B | Yes [68]; no [441] |  |
| Best quality of data |  | O | Binary [119]; Ordinal [62]; Quantitative [328] |  |
| Availability of data | Available to view | O | None [129]; report [80]; summary of data [149]; full dataset [151] |  |
|  | Available to download | O | None [325]; summary of data [106]; full dataset [78] |  |
|  | Available in real time | B | Yes [180]; no [328] |  |

* Data type: N=number, C=categorical, O=ordinal

**Table B.** **Results of the segmented regression of the increase in citizen science projects over time.** The most parsimonious model is highlighted in bold (assessed as the model with smallest degrees of freedom within 2 AIC units of the minimum). The results from this model are presented in Fig. 1 in the main text.

| Number of breakpoints | Degrees of freedom | AIC | ΔAIC | Point estimate of break points (years) |
| --- | --- | --- | --- | --- |
| **2** | **7** | **-149.1** | **0** | **1962, 1987** |
| 3 | 9 | -150.5 | -1.4 | 1962, 1988, 2012 |
| 1 | 5 | -67.5 | 81.6 | 1980 |

**Table C.** **The correlation of the individual attributes with the first seven factors of the multifactor analysis (MFA).**

|  |  | Factor 1 | Factor 2 | Factor 3 | Factor 4 | Factor 5 | Factor 6 | Factor 7 |
| --- | --- | --- | --- | --- | --- | --- | --- | --- |
| Percent of total variance explained |  | 12.9 | 8.8 | 7.8 | 5.8 | 5.0 | 4.7 | 4.0 |
| Cumulative percent of total variance explained |  | 12.9 | 21.7 | 29.5 | 35.4 | 40.4 | 45.1 | 49.1 |
| Attributes |  |  |  |  |  |  |  |  |
| Geographic scope |  | 0.57 | 0.18 | 0.00 | 0.49 | 0.26 | 0.02 | 0.15 |
| Aims |  | -0.25 | 0.15 | -0.21 | 0.07 | 0.39 | -0.12 | -0.42 |
| Background context |  | 0.03 | 0.48 | -0.16 | 0.11 | 0.38 | -0.03 | -0.22 |
| Protocol | Site selection by participant | 0.66 | 0.07 | 0.13 | 0.11 | 0.23 | -0.10 | 0.08 |
|  | One-off snapshots sufficient | 0.65 | -0.19 | 0.18 | -0.06 | -0.08 | -0.14 | -0.17 |
|  | Repeat visits required | -0.62 | 0.10 | -0.10 | 0.05 | -0.15 | 0.16 | -0.15 |
| Routes to engagement | Website | 0.27 | 0.47 | -0.20 | -0.14 | -0.16 | 0.11 | -0.16 |
|  | Smartphone | 0.36 | 0.03 | 0.32 | -0.06 | -0.19 | 0.07 | 0.00 |
|  | Social media | 0.15 | 0.12 | -0.02 | -0.12 | 0.24 | -0.09 | -0.06 |
|  | Mail | -0.15 | 0.01 | 0.13 | 0.16 | 0.22 | -0.22 | 0.18 |
|  | Email | -0.32 | -0.26 | 0.08 | 0.24 | 0.20 | -0.02 | -0.05 |
|  | SMS | 0.06 | 0.07 | 0.08 | 0.00 | 0.04 | -0.05 | -0.09 |
|  | Personal invitation | -0.46 | -0.15 | -0.09 | -0.30 | -0.12 | 0.04 | 0.12 |
| Type of support | Inadvance | -0.40 | -0.09 | -0.25 | 0.15 | -0.02 | 0.05 | -0.08 |
|  | Personal training | -0.69 | -0.09 | -0.12 | 0.01 | -0.02 | 0.04 | -0.14 |
|  | Supporting material | 0.29 | 0.52 | -0.07 | 0.00 | 0.20 | 0.07 | 0.11 |
|  | Online media | 0.06 | 0.34 | -0.13 | -0.01 | 0.27 | -0.12 | 0.01 |
| Target audience | Schoolchildren | 0.03 | 0.44 | -0.02 | -0.45 | 0.35 | 0.03 | 0.02 |
|  | Experts | -0.20 | -0.24 | 0.13 | 0.72 | 0.15 | -0.06 | -0.08 |
| Registration required |  | 0.02 | 0.36 | -0.37 | 0.23 | -0.18 | 0.60 | -0.18 |
| Entirely computer-based |  | 0.36 | -0.16 | -0.81 | 0.09 | -0.05 | -0.06 | 0.15 |
| Different types of data questions |  | -0.46 | 0.51 | -0.02 | -0.01 | 0.07 | -0.28 | 0.07 |
| Special equipment required |  | -0.62 | 0.10 | 0.10 | 0.13 | 0.20 | 0.28 | 0.27 |
| Type of record | Location | -0.28 | 0.16 | 0.77 | 0.06 | 0.04 | 0.00 | -0.27 |
|  | Photo | 0.35 | 0.23 | 0.38 | -0.08 | -0.09 | -0.04 | 0.09 |
|  | Score | -0.18 | 0.31 | -0.10 | 0.17 | -0.40 | -0.47 | -0.19 |
|  | OtherMedia | -0.05 | 0.05 | 0.02 | -0.16 | 0.01 | -0.04 | 0.60 |
|  | PhysicalSample | -0.31 | -0.19 | 0.01 | -0.22 | 0.39 | 0.40 | 0.16 |
| Best quality of data |  | -0.42 | 0.58 | -0.11 | 0.13 | -0.23 | -0.23 | 0.30 |
| Availability of data | To download | -0.11 | 0.26 | 0.15 | 0.15 | -0.30 | 0.22 | 0.13 |
|  | To view | 0.11 | 0.42 | 0.33 | 0.16 | -0.27 | 0.36 | 0.04 |
|  | In realtime | 0.34 | 0.34 | 0.38 | 0.16 | -0.15 | 0.27 | 0.08 |


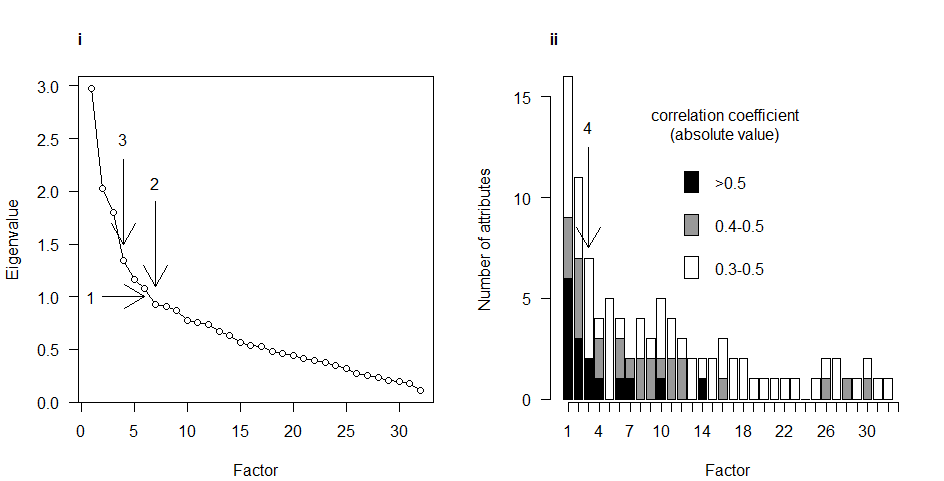


**Figure A.** **Assessment of the multifactor analysis (MFA) results according to (i) a scree plot of eigenvalues against factors and (ii) number of attributes correlated with factors.** The optimum number of factors to be considered varies according to the rule of thumb considered: (1) factors with eigenvalues greater than 1, (2) estimate of where the gradient of the scree plot changes, (3) estimate where the rate of decline of eigenvalues substantially slows and (4) the factors which are strongly correlated to more than one attribute. Overall, these suggest the optimum number of factors is between three and seven, and based on the results of Table C in S1 File, the conservative number of three factors is considered in this study.


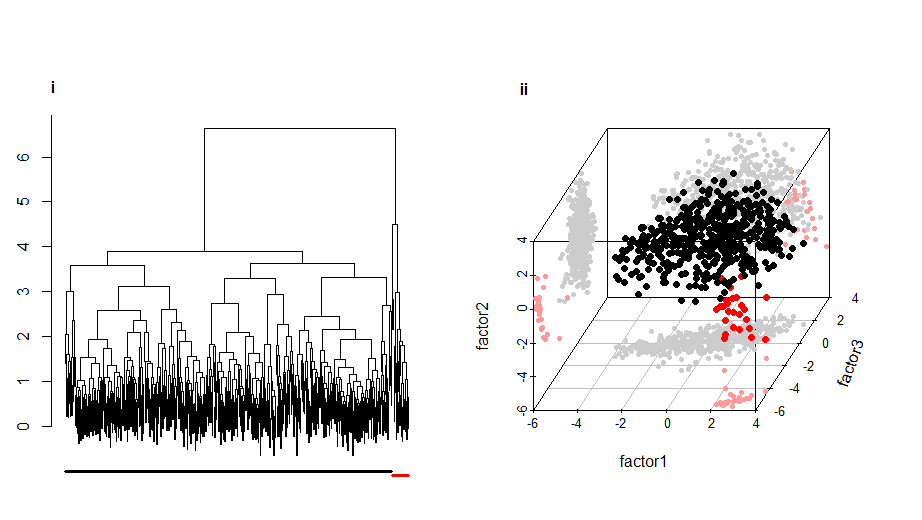


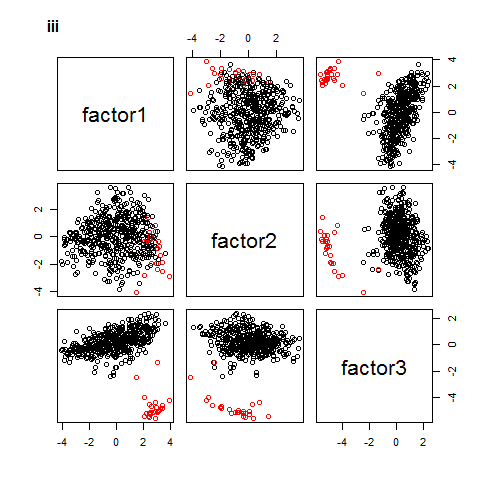


**Figure B.** **The distribution of projects along the first three factors from the multifactor analysis.** (i) Cluster analysis revealed two distinct clusters, shown in black and red, and this was due primarily to position along the third factor and shown in a three-dimensional plot (ii; points in three-dimensional space, with paler points indicating the projection on the three axes) and pairwise plots of the three factors (iii). Black and red points in (ii) and (iii) indicate the two clusters. The majority of projects, coloured black in these plots, are continuously distributed along factors 1 and 2 with no further clustering evident.


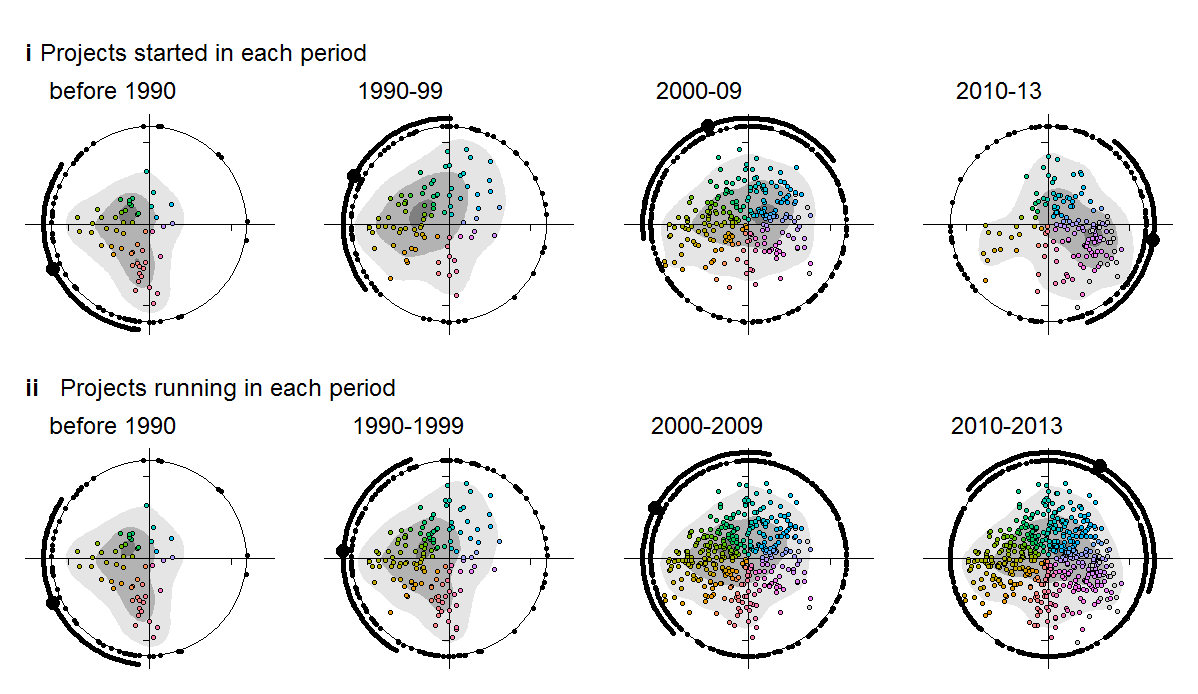


**Figure C.** **Variation in the kernel and calculation of angular position and deviation between the different time periods when considering projects according to (i) the time period in which they started (the ‘emerging diversity’) and (ii) the time periods in which they were active (the ‘accumulated diversity’).** The shifting focus over time is revealed by kernels (dark, mid and light grey indicating 5, 50 and 90% kernels, respectively) and angular position (individual projects projected as black points on the circle, with the arc showing angular mean ± deviation).


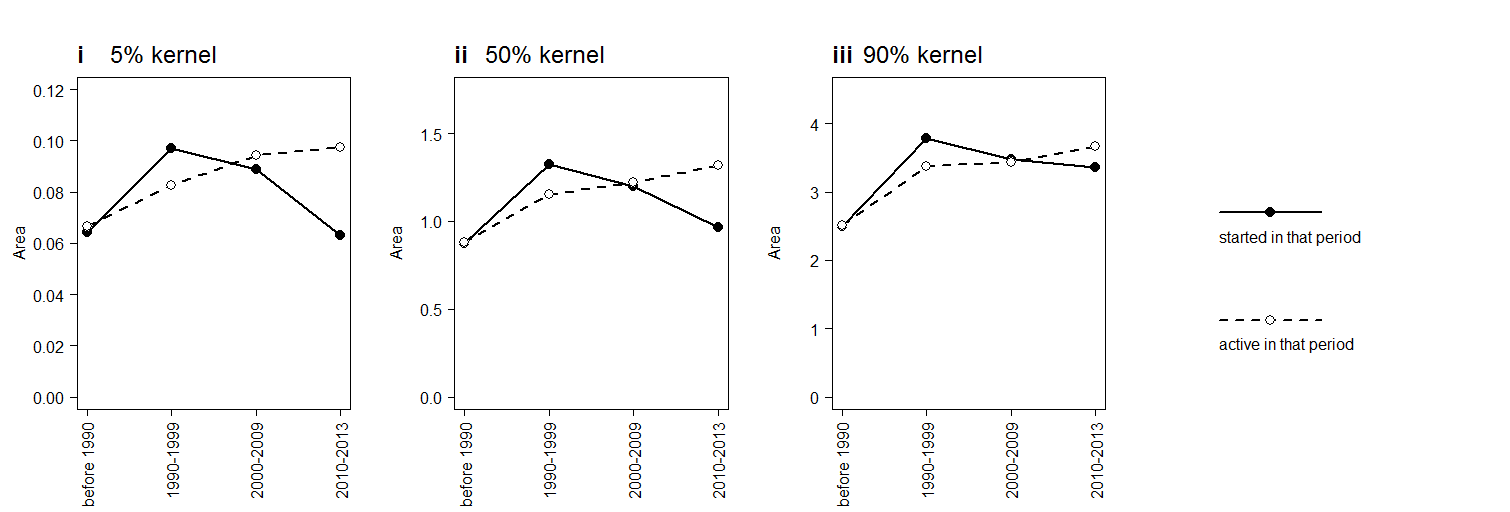


**Figure D.** **The area of the distribution of projects according to the first two factors of the multifactor analysis for different probabilities of the kernel for the emerging and the accumulated diversity (solid and dotted lines, respectively).** The pattern of values for the (i) 50% kernel (repeated from Fig. 3 G & H in the main text) are similar to those for the (ii) 5% kernel and (iii) 90% kernel. This demonstrates that our conclusion is robust to the selection of the kernel threshold. Overall, accumulated diversity (dotted line) shows a consistent increase over time, whereas the emerging diversity (the diversity of projects begun in each time period; solid line) does not increase over time.

**Appendix A.** Summary of analysis of additional supplementary variables.

In the main text we report on the analysis of the 32 explanatory variables and used the start and end period of each project as a supplementary variable. In addition to these we also assessed six more supplementary variables (each treated as categorical variables) as summarized in the table below.

**Table D.** Six supplementary attributes of the 509 projects in the analysis.

| Attribute | Options [number of projects] | Notes |
| --- | --- | --- |
| Main subject | Biodiversity [394]; environmental [115] | This is the main subject of the research in ‘ecology and the environment’. |
| Physical domain | Freshwater [102]; Marine [75]; Terrestrial [291]; Atmospheric [22]; Any [19] | This is the physical domain that is the primary subject of the research. |
| Project purpose | Biological recording [197];  biological monitoring [135];  environmental recording [29];  environmental monitoring [21];  water monitoring [54];  phenology [15];  hypothesis-led [15];  crowd-sourced [29];  technology platforms [14] | This is a classification of the purpose of the projects as obtained from publically-available information. We distinguished ‘recording’ (making a record) from ‘monitoring’ (which requires recording over a longer period). ‘Biological’ indicates that the purpose of the project was biodiversity, ‘environmental’ indicates the biophysical environment, and ‘water monitoring’ is a specific subset of ‘environmental monitoring’. ‘Phenology’ (recording seasonal timings of biological events) is a distinct set of projects for which the purpose was biological and environmental. Some projects clearly stated that their aims were to test hypotheses, and these were distinguished as ‘hypothesis-led’. The ‘entirely computer based’ projects were classified as ‘crowd-sourcing’ interpretation of data. Finally, some projects provide 'technology platforms’ to facilitate other projects. |
| Degree of participation | Contributory [474]; Collaborative [21]; Co-created [14] | According to the classification proposed by Bonney et al. in ref [23] |
| Project lead partner | Government [81], NGO (non-governmental organization) [248], Academic [167], Commercial [13] | ‘Government’ includes any national or local governmental organization; ‘academic’ includes research institutes |
| Project partners | 1 [296]; 2 [77]; 3-4 [79]; >4 [57] | Number of different organizations formally recognized as project partners |

The variables which were used as explanatory variables in the main text were selected because they explained how the project was run. The supplementary variables reported here (and the time periods in which each project started and was active, as reported in the main text), provide further description about the subject of the project and so we can show how the attributes correspond to the results of the multi-factor analysis (MFA). For some of these attributes we accept that it was harder to unequivocally classify the projects (especially degree of participation and project purpose; the latter of which was categorized post hoc rather than a priori). The correspondence of the supplementary attributes to the results of the multivariate analysis is shown in Fig. E.

Care needs to be taken in interpreting the project attributes shown in Fig. E because they could be strongly associated with each other. For example, there are many projects about freshwaters that are established to undertake long-term monitoring and led by local governments. It may be that different attributes influence project approaches in complex ways, for example, it may be that the growth of academic involvement in citizen science, which was encouraged by the agenda for public engagement in science, drove a move towards more elaborate, mass participation approaches in 2000-9. Clearly the development of citizen science is a complex phenomenon influenced by many different stakeholders: organisers, participants, society, policy and funders.

**Figure E.** Distribution of projects according to categories of the six supplementary variables shown in Table D in File S1. The location of each project on the first two axes of the multiple factor analysis is shown as white dots, with the axes as shown in Fig. 2 of the main paper. The distributions of the points are summarized by the underlying kernels of the distributions (dark orange, light orange and grey showing the 90%, 50% and 5% kernels, respectively). Categories are ordered according their position on the first (horizontal) axis, except for the number of project partners which is numerically ordered.

**i.** Main subject


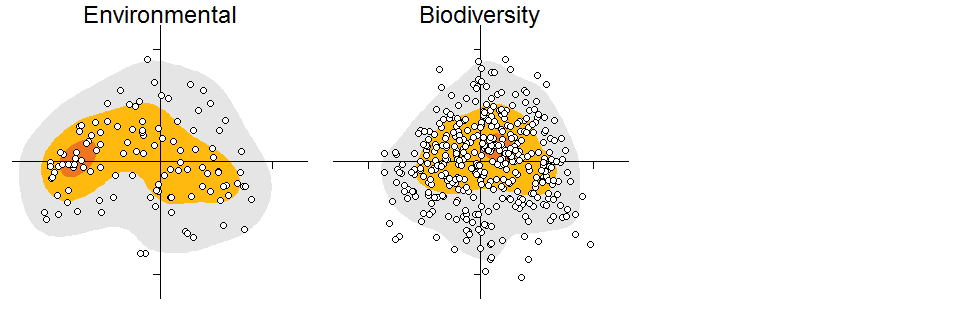


**ii.** Physical domain


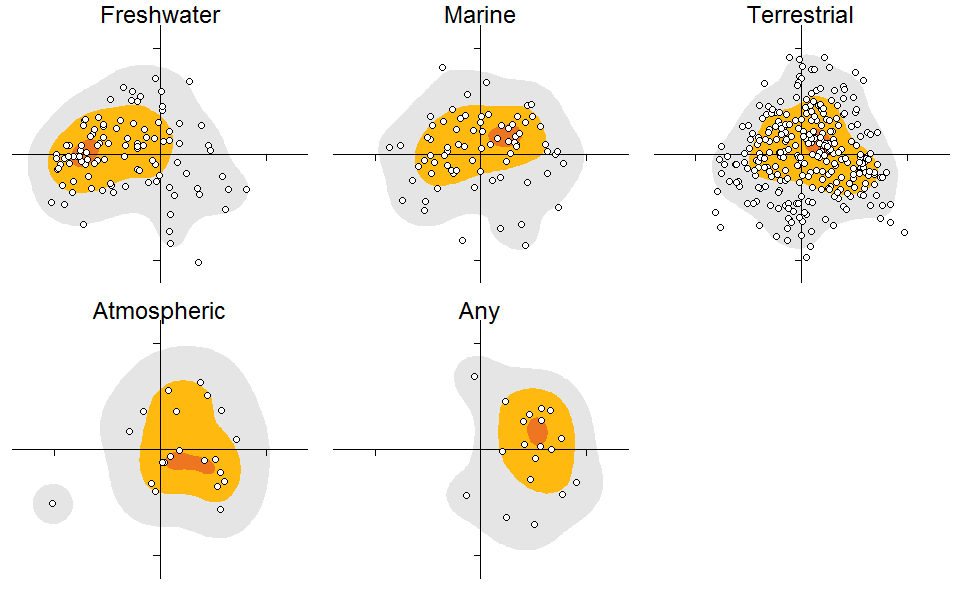


**iii.** Project purpose


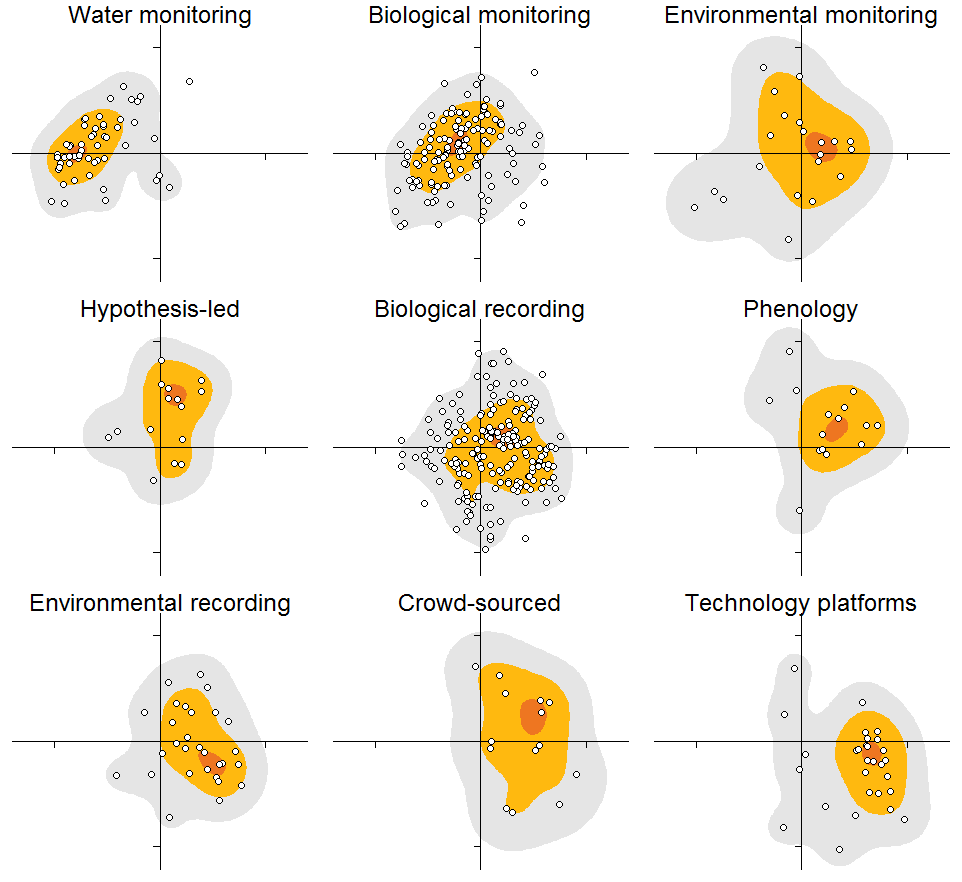


**iv.** Degree of participation


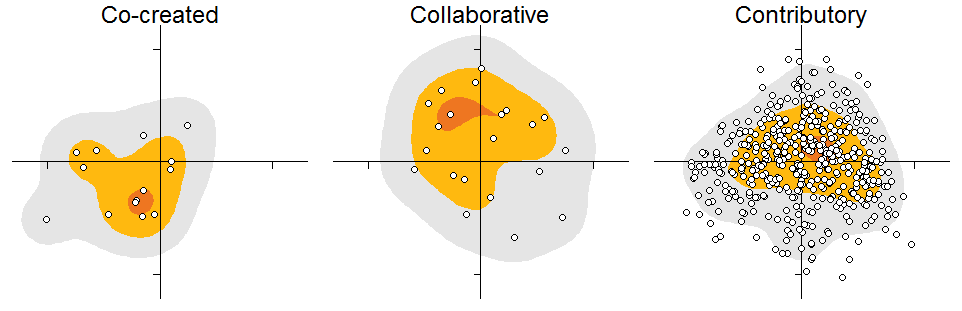


**v.** Project lead partner


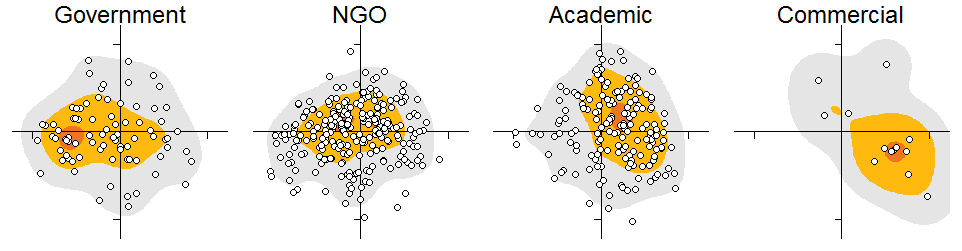


**vi.** Number of project partners


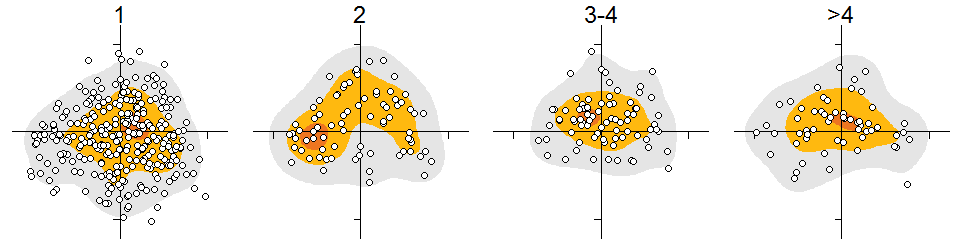

Supplement: S1 File — Supplementary information containing: Table A. The 32 attributes (and associated subcategories) used for scoring the 509 projects. Table B. Results of the segmented regression of the increase in citizen science projects over time. Table C. The correlation of the individual attributes with the first seven factors of the multifactor analysis (MFA). Figure A. Assessment of the multifactor analysis (MFA) results according to (i) a scree plot of eigenvalues against factors and (ii) number of attributes correlated with factors. Figure B. The distribution of projects in the first three factors from the results of the multifactor analysis. Figure C. Variation in the kernel approach and calculation of angular position and deviation between the different time periods when considering projects according to (i) the time period in which they started (the ‘emerging diversity’) and (ii) the time periods in which they were active (the ‘accumulated diversity’). Figure D. The area of the distribution of projects according to the first two factors of the multifactor analysis for different probabilities of the kernel for the emerging and the accumulated diversity (solid and dotted lines, respectively). Appendix A. Summary of analysis of additional supplementary variables: main subject, physical domain, project purpose, degree of participation, type of project lead partner and number of project partners. This includes Table D and Fig. E. (DOCX) [file pone.0172579.s001.docx]
